# Preterm Birth: A Prominent Risk Factor for Low Apgar Scores

**DOI:** 10.1155/2015/978079

**Published:** 2015-08-27

**Authors:** Maria Svenvik, Lars Brudin, Marie Blomberg

**Affiliations:** ^1^Department of Obstetrics and Gynecology, Kalmar County Hospital, 391 85 Kalmar, Sweden; ^2^Department of Clinical Physiology, Kalmar County Hospital, 391 85 Kalmar, Sweden; ^3^Department of Medicine and Health Sciences, Linköping University Hospital, 581 85 Linköping, Sweden; ^4^Department of Obstetrics and Gynecology, and Department of Clinical and Experimental Medicine, Linköping University, 581 83 Linköping, Sweden

## Abstract

*Objective.* To determine predictive risk factors for Apgar scores < 7 at 5 minutes at two hospitals providing tertiary care and secondary care, respectively. *Methods.* A retrospective registry cohort study of 21126 births (2006–2010) using data from digital medical records. Risk factors were analyzed by logistic regression analyses. *Results.*  AS^5min⁡^ < 7 was multivariately associated with the following: preterm birth; gestational week 32 + 0–36 + 6, OR = 3.9 (95% CI 2.9–5.3); week 28 + 0–31 + 6, OR = 8 (5–12); week < 28 + 0, OR = 15 (8–29); postterm birth, OR = 2.0 (1.7–2.3); multiple pregnancy, OR = 3.53 (1.79–6.96); previous cesarean section, OR = 3.67 (2.31–5.81); BMI 25–29, OR = 1.30 (1.09–1.55); BMI ≥ 30  OR = 1.70 (1.20–2.41); nonnormal CTG at admission, OR = 1.98 (1.48–2.66). ≥1-para was associated with a decreased risk for AS^5min⁡^ < 7, OR = 0.34 (0.25–0.47). In the univariate logistic regression analysis AS^5min⁡^ < 7 was associated with tertiary level care, OR = 1.48 (1.17–1.87); however, in the multivariate analysis there was no significant difference. *Conclusion.* A number of partially preventable risk factors were identified, preterm birth being the most evident. Further, no significant difference between the two hospital levels regarding the risk for low Apgar scores was detected.

## 1. Introduction

The Apgar score, regardless of underlying cause, is used for comparing the neonatal outcome at different obstetrical units worldwide in order to measure the quality of obstetrical care. The Apgar score still defines the degree of birth asphyxia according to the International Statistical Classification of Diseases and Related Health Problems 10th Revision (ICD-10) [1], despite other available methods indicating birth asphyxia such as umbilical cord acid base balance measurement.

A low Apgar score less than seven points at five minutes is known to have implications for neonatal mortality [2] and morbidity, for example, respiratory distress and neurological problems [3, 4]. Still, the vast majority of infants with Apgar < 7 points at five minutes (AS^5 min^ < 7) will be healthy both during the neonatal period and later on in life.

Previous national and international studies have demonstrated a number of risk factors for low Apgar scores or asphyxia, although results and definitions differ. The studies comprise socioeconomic, demographic, and medical risk factors. Smoking [5], low socioeconomic status [6], single civil status of the mother [7], maternal short stature [8, 9], and maternal obesity [10, 11] have all been shown to increase the risk for a low Apgar score. The mode of delivery, intrauterine meconium release, and abnormalities in cardiotocography (CTG) [7] comprise medical risk factors associated with a low Apgar score. There are no available comprehensive studies evaluating the state of modern Swedish obstetric health care.

The aims of the study were to investigate whether there are identifiable risk factors of AS^5 min^ < 7 prior to delivery and to compare possible differences in risk factor profiles between the level of care at a university hospital providing tertiary care and a county hospital providing secondary care.

## 2. Materials and Methods

This retrospective register study is a comprehensive study of all 21 126 deliveries during five consecutive years (2006–2010) at a secondary level hospital (the County Hospital in Kalmar) and a tertiary level hospital (the University Hospital in Linköping), both situated in the southeastern region of Sweden. Parturients and infants were identified from the local digital medical records, Obstetrix (Siemens Healthcare, Health Services, Upplands Väsby, Sweden),which include standardized medical record forms completed at the antenatal health care centers at the start of prenatal care (usually in gestational week 10–12), records from the delivery units, and records from the pediatric examination of the newborn. Ninety percent visit the antenatal health care center during the first trimester of pregnancy (local data). The system is identical at both hospitals.

The study population was divided into infants with an AS^5 min^ < 7 and infants with an Apgar score ≥ 7 at five minutes (AS^5 min^ ≥ 7). Infants, who had not been given an Apgar score at five minutes were excluded (*n* = 136). Stillbirths due to intrauterine fetal death (IUFD) were also excluded (*n* = 72), as were infants with lethal malformations (*n* = 3; anencephaly, Potter syndrome and trisomy 18).

In cases of multiple pregnancies (*n* = 619) one of twins or two of triplets (*n* = 6) were excluded in order to avoid recording potential maternal risk factors more than once in the same pregnancy. Among siblings where one had an AS^5 min^ < 7 while the other had an AS^5 min^ ≥ 7, those with AS^5 min^ < 7 were included in the study. When both infants in a pair of twins had an AS^5 min^ < 7 or both had an AS^5 min^ ≥ 7, one was randomly excluded.

Due to partial overlapping (e.g., twin and IUFD) in the mentioned exclusion criteria 483 infants were excluded. A total of 20 643 (97.7%) patients were available for further analysis.

From the medical records data was extracted regarding maternal age, height, and weight, single or multiple pregnancy, parity, smoking habits during pregnancy, previous cesarean section, previous or current illnesses in certain categories (type 1 diabetes mellitus, endocrine diseases (i.e., not diabetes mellitus, predominantly thyroid disease), epilepsy and inflammatory bowel disease (IBD)), induction versus spontaneous onset of labor, classification of the CTG at admission, gestational age at delivery, mode of delivery, time of delivery, and diagnoses at delivery including preeclampsia if existent.

Body mass index (BMI) in kg/m^2^ was calculated from maternal prepregnancy weight and height data. Women were grouped into six categories of BMI: underweight (less than 18.5), normal weight (18.5–24.9), overweight (25–29.9), obesity class I (30–34.9), obesity class II (35–39.9), and obesity class III (40 or more) according to the World Health Organization classification [12]. All these maternal and delivery variables were considered potential risk factors for low Apgar scores and were compared between infants with AS^5 min^ < 7 and infants with AS^5 min^ ≥ 7. Secondly, these variables were compared concerning infants with AS^5 min^ < 7 and infants with AS^5 min^ ≥ 7 between the two hospital levels included in the study.

Continuous variables with symmetric distribution (e.g., age and weight) are presented as the mean value (mean), standard deviation (SD) and range (min–max), and categorized for logistic regression analysis. The difference between groups with respect to these variables was analyzed with Student's *t*-test adjusted for unequal variances. Risk factors for AS^5 min^ < 7 were analyzed by logistic regression analysis. Significant associations of AS^5 min^ < 7 in the univariate analysis and age were included in the subsequent multivariate logistic regression analysis. In this analysis, data is presented as odds ratios (OR) with 95% confidence intervals (CI). All *P* values are two-tailed and *P* = 0.05 is considered statistically significant. The software Statistica v.12 (StatSoft, Inc., Tulsa, OK, USA) was used for all analyses.

The study was approved by the Regional Ethical Review Board in Linköping, Sweden (2012/195-31).

## 3. Results

Of 21126 infants 20990 (99.4%) had been assigned an Apgar score at five minutes of age. After described exclusions a total number of 20643 were considered the study population. From the tertiary level hospital 13632 infants were included, of which 273 had AS^5 min^ < 7 (2.00%). From the secondary level hospital, the corresponding figures were 96/7011 (1.37%).

There was no difference in maternal age or maternal weight between the group with an AS^5 min^ < 7 and an AS^5 min^ ≥ 7 (Table 1), but maternal height was significantly shorter in the group with AS^5 min^ < 7. The BMI was significantly higher in the group with AS^5 min^ < 7 and there was a clear trend over the BMI strata as well as for gestational age (Table 2). The mean gestational age was significantly shorter in AS^5 min^ < 7 (Table 1).

AS^5 min^ < 7 was multivariately associated with the following parameters (Table 3): preterm birth, postterm birth, multiple pregnancy, previous cesarean section, preeclampsia, maternal height ≤ 158 cm, BMI 25–29, and BMI ≥ 30. Being ≥ 1-para was associated with a decreased risk for AS^5 min^ < 7. In the univariate logistic regression analysis AS^5 min^ < 7 was associated with tertiary level care. However, in the multivariate analysis no significant difference was found. This was also the case for thyroid disease. Neither type 1 diabetes mellitus, IBD, nor epilepsy was significant risk factors for AS^5 min^ < 7.

Analyses were also performed to investigate whether or not the time of birth (day/night or seasonal) was a risk factor for a low Apgar score, but no significant difference was found.

Furthermore, different types of delivery modes were investigated for the frequency of low Apgar scores. There was no significant difference between spontaneous vaginal birth (0.91% AS^5 min^ < 7) and elective cesarean section (1.24%) or between operative vaginal delivery (4.68%) and emergency cesarean section (5.70%). In the group delivered by immediate emergency cesarean section, however, there was a significantly higher frequency of AS^5 min^ < 7 (20.9%, *P* < 0.001).

Moreover, a subanalysis was performed in order to investigate risk factors in the study population for birth before 32 weeks of gestation (Table 4), which was multivariately associated with multiple pregnancy, preeclampsia, smoking, thyroid disease, and epilepsy. Being ≥ 1-para was associated with a decreased risk for birth before 32 weeks of gestation.

## 4. Discussion

This retrospective registry study showed that maternal height < 158 cm, BMI > 30 kg/m^2^, nulliparity, postterm birth (≥42 weeks of gestation), preterm birth (≤36 + 6 weeks of gestation), previous cesarean section, multiple pregnancy, preeclampsia, and an abnormal CTG at admission were independent risk factors for AS^5 min^ < 7.

The study has some limitations. One is that the cases of AS^5 min⁡^ < 7 are relatively few, as they constitute 1.98% of the total study population. This could be rectified by a larger study population. As the number of children with an AS^5 min^ ≥ 7, however, is relatively large, the risk factor analysis should be considered fairly robust.

A low Apgar score does not always correlate to neonatal asphyxia on the basis of metabolic acidosis. The analysis of umbilical cord pH and base excess was not included in this study, which could have been valuable in order to further elucidate the entity of infants with low Apgar scores. However, it has been previously shown that only about 38% of children with low Apgar scores have metabolic acidosis [13]. In another study 69% of children with an Apgar score of 1–3 at 5 minutes and 54% of children with an Apgar score of 4–6 at 5 minutes had a pH of <7.15 in the umbilical artery [14]. On the other hand, not all neonates with metabolic acidosis have low Apgar scores [14, 15]. Although the pathophysiology of low Apgar scores differ, a low score identifies children in need of resuscitation efforts at birth. An advantage of using the Apgar score as a selection criterion, and not metabolic acidosis, is that the vast majority of infants were given an Apgar score (in this material 20 990/21 126 = 99.4%) whereas complete registration of umbilical cord blood gases were at hand for only about 70–75%, thereby missing about 25–30% (local data).

In this study we chose to include all live births during the relevant time period at two hospitals. We did not set a minimum threshold for inclusion regarding gestational age, which was often the case in other studies aiming to investigate risk factors for low Apgar scores, neonatal asphyxia, or hypoxic ischemic encephalopathy [7, 10, 16]. We also chose not to exclude cases of multiple gestation, as the purpose was to comprehensively investigate risk factors for AS^5 min^ < 7. In similar studies exclusively singleton pregnancies were included [7, 16]. Our study, therefore, presents a more genuine view of the risk factors for low Apgar scores among patients at a standard Swedish obstetric unit.

This study focused on maternal risk factors and only those easily accessible in the digital medical journals. One advantage of using medical journals as a data source is that this enables including variables not available in the Swedish Medical Birth register. Here, for example, we have the possibility to evaluate the CTG at admission. This is a strength of the study.

Regarding fetal risk factors, we have studied the impact of gestational age and normal or abnormal CTG at admission. In this kind of study it is not possible, however, to further evaluate CTG or the use of an ST-analysis. It would be of value to investigate other potential fetal risk factors, such as intrauterine growth restriction or oligohydramniosis. However, we estimated the material as too small for this purpose. Another problem with those factors is that they may not have been diagnosed prior to delivery.

Although height and BMI are so closely linked, maternal short stature (≤158 cm) and BMI > 30 kg/m^2^ were both found to be independent risk factors for AS^5 min^ < 7, even when adjusted for in the multivariate regression analysis. However, information on height and/or weight was lacking in 2103 individuals (10.2%), of which 68 were in the group AS^5 min^ < 7 (3.2%), which is a slight overrepresentation and implies that the results regarding BMI as a risk factor might have to be interpreted with some caution, although this has been verified in previous studies [10, 11]. It is interesting to note that maternal height is of such importance, as this appears to be an empirical observation made by experienced midwives and obstetricians. This has also previously been shown both nationally [16] and internationally [8, 9], although the latter studies are older. The risk factors nulliparity [10, 16] and previous cesarean section [16] are also in line with the results of other studies. One must bear in mind, however, that the comparison of risk factors in an international perspective might be difficult, as antenatal care as well as obstetric care is conducted under completely different conditions in large parts of the world compared to the southeastern region of Sweden.

Concerning the frequency of low Apgar scores in relation to different modes of delivery we have looked at the frequency per se, since there was no possibility in this kind of study to evaluate indications, for example, a pathological CTG pattern for operative vaginal delivery or cesarean section. It might be valuable to investigate this, since a Swedish study showed that two-thirds of children born after 33 weeks of gestation with an AS^5 min^ < 7 were subject to substandard care during labor, where the most common causes were misinterpretation of CTG, incorrect action based on CTG changes, and nonoptimal use of oxytocin augmentation of labor [17].

We chose to compare two obstetric clinics at a secondary and a tertiary level hospital in the same region of Sweden. There is an established collaboration between the two clinics and obstetric high-risk patients are, in some instances, referred from the secondary level hospital to the tertiary level hospital. From this perspective, risk factors such as differences in obstetric care are important to continually analyze and evaluate. In the univariate logistic regression analysis tertiary level of care was a slight risk factor for low Apgar scores. However, when adjusted for other parameters, such as preterm birth, there was no significant difference regarding the risk profile for AS^5 min^ < 7 between the two levels of hospital care.

The most evident risk factor for AS^5 min^ < 7 found in this study was preterm birth (OR = 8 (5–12) and OR = 15 (8–29) for gestational age 28 + 0–31 + 6 weeks and <28 + 0 weeks, resp.).

The overall rate of preterm birth before 37 weeks of gestation in this population was 7.74%, compared to 5.9% in the general Swedish population [18]. The reason for the slightly higher incidence of preterm birth in the study population was the referral of preterm deliveries to the tertiary care hospital. In an international perspective the incidence of preterm birth in Sweden is low, compared to, for example, the United States, where preterm birth rates are 12% [18]. The frequency of preterm births before 32 weeks of gestation was low, even at the tertiary level hospital (1.7%). The total study population included 260 (1.26%) infants born before 32 weeks of gestation, of which 89 (34%) had an AS^5 min^ < 7. In comparison, the frequency of AS^5 min^ < 7 among term infants (week 37 + 0–41 + 6) was only 1.1%. It has also been previously shown that a very low gestational age and a very low birth weight correlate to low Apgar scores [19].

Since preterm birth was found to be such a strong risk factor for AS^5 min^ < 7, we chose to investigate this issue further with the material at hand. We found multiple pregnancy to be a very strong factor for preterm birth (OR = 15 (9–24)), which has been previously confirmed [20]. When dealing with in vitro fertilization, for example, it is, therefore, of vital importance to reduce the risk of multiple pregnancy in order to avoid the risk of preterm birth, which accounts for the vast majority of neonatal morbidity and mortality [21]. Preeclampsia was also found to be a significant risk factor (OR = 5.5 (3.4–8.9)) for birth before 32 weeks of gestation, although this was anticipated since severe preeclampsia is a cause of medically indicated preterm birth. Previous studies have shown various causes of preterm birth [22], and in line with these results we demonstrated smoking as a risk factor for birth before 32 weeks of gestation, implicating the need for continuous work in the field of health informatics. In a Cochrane review by Chamberlain et al. [23] it was demonstrated that psychosocial interventions for the cessation of smoking during pregnancy were successful, and led to a lower frequency of preterm births. We also found thyroid disease and epilepsy to be independent significant risk factors for birth before 32 weeks of gestation. The number of cases of maternal thyroid disease are relatively few (*n* = 550, of which only 10 infants were born before 32 weeks) and the data does not differ between different types of thyroid dysfunction. However, the function of the thyroid gland is central in human reproduction and untreated thyroid disorders are known to increase the risk of several adverse outcomes in pregnancy, of which preterm birth is one [24]. Furthermore, we showed that parturients who were ≥1-para, had a decreased risk for preterm birth implying the need for more thorough monitoring of pregnancies in primigravidae.

Although this project provided valuable baseline data, there is reason to pursue these analyses on a regular basis, to detect trends, for example, in frequency of cesarean sections, inductions, and instrumental vaginal deliveries. Furthermore, the average BMI among pregnant women, as well as the proportion of obese pregnant women, has been shown to be increasing [25, 26]. This is the single risk factor for low Apgar scores shown in this study possible to influence, which stresses the importance of working intensely with this issue to reduce obesity and call attention to associated risks, not only regarding pregnancy outcome but regarding cardiovascular and metabolic diseases. These results may constitute the basis for intervention studies such as those conducted on obese mothers in Linköping, Sweden [27]. Although this study could not demonstrate obesity as a risk factor for preterm birth, meta-analyses show an association between these two conditions [28]. If interventions could lead to a reduced degree of obesity among pregnant women, we might see an effect on the frequency of preterm birth and thereby a lower incidence of low Apgar scores as well.

## 5. Conclusion

Preterm birth is the most evident risk factor for low Apgar scores, and interventions to prevent preterm birth are of importance in improving neonatal outcome. From a clinical perspective efforts are needed to reduce maternal obesity and smoking, since these two risk factors are possible to influence.

## Figures and Tables

**Table 1:** Descriptive data for the study population (*n* = 20 643). Differences between AS^5 min^ < 7 and AS^5 min^ ≥ 7 were analyzed with Student's *t*-test.

	AS^5 min^ < 7			AS^5 min^ ≥ 7			*P*
	*n*	Mean (SD)	Q1–Q3	*n*	Mean (SD)	Q1–Q3	
Age (years)	369	30 (5)	26–34	20274	30 (5)	26–33	0.891
Weight (kg)	303	70 (15)	59–78	18314	69 (14)	59–75	0.133
Height (cm)	306	164 (7)	160–168	18570	166 (6)	162–170	<0.001
Body mass index (kg/m²)	301	26 (5)	22–29	18239	25 (5)	22–27	<0.001
Gestational age (weeks)	366	36 (6)	32–41	20251	40 (2)	39–41	<0.001

**Table 2:** Categorized descriptive data for the study population regarding body mass index (BMI) and gestational age. Chi2-test was used for the comparison of frequencies.

Parameter	AS^5 min^ < 7		AS^5 min^ ≥ 7		*P*
	*n*	(%)	*n*	(%)	
BMI (kg/m^2^)					
<20	21	7.0	1746	9.6	
20–24.9	135	44.9	9457	51.9	
25–29.9	84	27.9	4778	26.2	
30–34.9	46	15.3	1566	8.6	
≥35	15	5.0	692	3.8	<0.001^*^
Total	**301**	**100**	**18239**	**100**	
Gestational age (weeks)					
<28	62	16.9	49	0.2	
28–31 + 6	27	7.4	122	0.6	
32–36 + 6	45	12.3	981	4.8	
37–41 + 6	206	56.3	18045	89.1	
≥42	26	7.1	1054	5.2	<0.001
Total	**366**	**100**	**20251**	**100**	

^*^Sign difference between BMI ≥ 30 and BMI < 30 (*P* < 0.001).

**Table 3:** Risk factors for Apgar scores < 7 at 5 minutes. Analyses performed by univariate and multivariate logistic regression.

	Total	AS^5 min^ < 7		Univariate logistic regression			Multivariate logistic regression		
		*n*	(%)	OR	95% conf. int.	*P*	OR	95% conf. int.	*P*
Level of care									
Secondary	7011	96	1.37	1.00					
Tertiary	13632	273	2.00	1.48	1.17–1.87	0.001	—	—	—
Age (years)									
≤26	5387	100	1.86	1.00			1.00		
27–30	5976	102	1.71	1.01	0.92–1.10		1.10	0.97–1.24	
31–33	4343	72	1.66	1.01	0.84–1.22		1.20	0.93–1.54	
≥34	4937	95	1.92	1.02	0.77–1.34	0.897	1.31	0.90–1.91	0.152
Height (cms)									
≥175	1800	17	0.94	1.00			1.00		
159–174	14992	229	1.53	1.83	1.44–2.32		2.06	1.56–2.72	
≤158	2084	60	2.88	3.34	2.06–5.39	<0.001	4.23	2.43–7.39	<0.001
BMI (kg/m^2^)									
<25	11359	156	1.37	1.00			1.00		
25–29.9	4862	84	1.73	1.38	1.19–1.60		1.29	1.08–1.54	
≥30	2319	61	2.63	1.91	1.43–2.56	<0.001	1.67	1.17–2.36	0.004
Smoking									
No	15784	246	1.56	1.00					
Yes	3236	62	1.92	1.25	0.95–1.66	0.113	—	—	—
Thyroid disease									
No	18379	290	1.58	1.00					
Yes	550	17	3.09	1.99	1.21–3.27	0.007	—	—	—
Type 1 diabetes mellitus									
No	18823	302	1.60	1.00					
Yes	134	5	3.73	1.21	0.97–5.85	0.059	—	—	—
Inflammatory bowel disease									
No	18762	302	1.61	1.00					
Yes	194	5	2.58	1.62	0.66–3.96	0.292	—	—	—
Epilepsy									
No	18798	303	1.61	1.00					
Yes	158	4	2.53	1.59	0.58–4.30	0.366	—	—	—
Parity									
0	8418	191	2.27	1.00			1.00		
≥1	10602	117	1.10	0.48	0.38–0.60	<0.001	0.34	0.25–0.48	<0.001
Gestational age (weeks)									
37 + 0–41 + 6	18251	206	1.13	1.00			1.00		
≥42 + 0	1080	26	2.41	2.8	2.6–3.1		2.0	1.7–2.3	
32 + 0–36 + 6	1026	45	4.39	8.0	6.8–9.4		3.9	2.9–5.3	
28 + 0–31 + 6	149	27	18.1	23	18–29		8	5–12	
<28 + 0	111	62	55.9	64	46–88	<0.001	15	8–29	<0.001
Previous cesarean section									
No	17796	271	1.52	1.00			1.00		
Yes	1224	37	3.02	2.01	1.42–2.84	<0.001	3.66	2.31–5.81	<0.001
Multiple pregnancy									
No	20338	336	1.65	1.00			1.00		
Yes	305	33	10.82	7.20	4.95–10.49	<0.001	3.57	1.81–7.05	<0.001
Preeclampsia									
No	20092	334	1.66	1.00					
Yes	551	35	6.35	4.01	2.80–5.74	<0.001	—	—	—
Induction									
No	18067	299	1.65	1.00					
Yes	2576	70	2.72	1.65	1.27–2.15	<0.001	—	—	—
CTG at admission									
Normal	16524	214	1.30	1.00			1.00		
Nonnormal	1176	36	3.06	2.47	1.73–3.53	<0.001	1.95	1.45–2.62	<0.001

**Table 4:** Risk factors for preterm birth before 32 weeks of gestation. Analyses performed by univariate and multivariate logistic regression.

Parameter	Total	32 weeks		Univariate logistic regression			Multivariate logistic regression		
		*n*	(%)	OR	95% conf. int.	*P*	OR	95% conf. int.	*P*
Age (years)									
≤26	5386	96	1.78	1.00			1.00		
27–30	5975	47	0.79	0.92	0.82–1.03		0.98	0.84–1.16	
31–33	4341	54	1.24	0.84	0.68–1.05		0.97	0.70–1.34	
≥34	4915	63	1.28	0.78	0.56–1.08	0.135	0.95	0.59–1.54	0.84
Height (cms)									
≥175	1800	18	1.00	1.00					
159–174	14992	99	0.66	1.11	0.77–1.59				
≤158	2084	23	1.10	1.22	0.59–2.54	0.588	—	—	—
BMI (kg/m^2^)									
<25	11359	80	0.70	1.00					
25–29.9	4862	39	0.80	1.09	0.87–1.38				
≥30	2319	19	0.82	1.19	0.75–1.89	0.448	—	—	—
Smoking									
No	15784	108	0.68	1.00			1.00		
Yes	3236	34	1.05	1.54	1.05–2.27	0.029	1.61	1.07–2.41	0.022
Thyroid disease									
No	18379	131	0.71	1.00			1.00		
Yes	550	10	1.82	2.58	1.35–4.93	0.004	2.32	1.17–4.57	0.015
Type 1 diabetes mellitus									
No	18823	134	0.71	1.00					
Yes	140	1	0.71	1.00	0.16–6.28	0.997	—	—	—
Inflammatory bowel disease									
No	18762	194	1.03	1.00					
Yes	138	3	2.17	2.12	0.67–6.71	0.201	—	—	—
Epilepsy									
No	18798	137	0.73	1.00			1.00		
Yes	158	4	2.53	3.54	1.29–9.67	0.014	3.14	1.13–8.78	0.029
Parity									
0	8418	82	0.97	1.00			1.00		
≥1	10602	60	0.57	0.58	0.41–0.81	0.001	0.68	0.47–0.98	0.036
Previous cesarean section									
No	17796	132	0.74	1.00					
Yes	1224	10	0.82	1.10	0.58–2.10	0.768	—	—	—
Multiple pregnancy									
No	20314	216	1.06	1.00			1.00		
Yes	303	44	14.52	15.8	11.2–22.3	<0.001	15	10–24	<0.001
Preeclampsia									
No	20067	214	1.07	1.00			1.00		
Yes	550	46	8.36	8.47	6.09–11.78	<0.001	5.48	3.39–8.86	<0.001
